# Trends in Outpatient Antibiotic Prescriptions Issued in Croatian Primary Healthcare, 2015–2024

**DOI:** 10.3390/idr18020036

**Published:** 2026-04-14

**Authors:** Anamaria Jurčević, Jelena Dimnjaković, Rok Čivljak

**Affiliations:** 1 Medical Informatics and Biostatistics Division, Croatian Institute of Public Health, 10000 Zagreb, Croatia; jelena.dimnjakovic@hzjz.hr; 2 Division for Respiratory Infections, University Hospital for Infectious Diseases “Dr. Fran Mihaljević”, 10000 Zagreb, Croatia; rcivljak@bfm.hr; 3 Department for Infectious Diseases, School of Medicine, University of Zagreb, 10000 Zagreb, Croatia

**Keywords:** antibiotic prescribing, primary healthcare, prescription trends, antimicrobial stewardship, AWaRe classification, Access antibiotics

## Abstract

Objectives: Outpatient antibiotic prescribing is a major driver of antimicrobial resistance, yet detailed long-term analyses of prescribing patterns in Croatia remain limited. This study aimed to analyze trends in outpatient antibiotic prescriptions issued in Croatian primary healthcare from 2015 to 2024, stratified by antibiotic class, substance, and the WHO AWaRe classification. Methods: A retrospective analysis of nationwide data on antibiotic prescriptions issued in primary care outpatient settings was conducted using the data from the Central Health Information System of the Republic of Croatia. All prescriptions for ATC group J01 antibiotics issued between 1 January 2015 and 31 December 2024 were included. The primary outcome was the annual number of issued outpatient antibiotic prescriptions, described overall and by substance. Annual counts were additionally expressed as a percentage of the 2015 baseline (index year = 100%) to enable the comparison across substances with different prescribing volumes. The prescriptions were classified according to the WHO AWaRe framework. Results: A total of 31,048,414 outpatient antibiotic prescriptions were issued between 2015 and 2024. Overall prescribing declined by 5.6% from 2015 to 2019, followed by a marked decrease of 21.0% in 2020, and subsequently rebounded to 3,338,235 prescriptions by 2024, a number virtually identical to pre-pandemic levels. Co-amoxiclav and azithromycin together accounted for 49.5% of all prescriptions. By 2024, prescribing third-generation cephalosporins increased by 281.9% compared to the 2015 levels, while prescribing amoxicillin decreased by 43.6% over the same period. The proportion of Access antibiotics declined from 64.7% in 2015 to 57.9% in 2024. Conclusions: The main challenge for antimicrobial stewardship in Croatia lies not only in overall prescribing volume but in prescribing composition. Targeted interventions are needed to reduce reliance on broad-spectrum agents and promote the use of narrower-spectrum first-line alternatives.

## 1. Introduction

Antimicrobial resistance (AMR) is a major global public health threat associated with substantial mortality and disability. The Global Burden of Bacterial AMR study estimated that, in 2019, 4.95 million deaths were associated with bacterial AMR and 1.27 million deaths were directly attributable to it [1]. In Croatia, the estimated burden in the same year was 2546 deaths and 46,958 disability-adjusted life years (DALYs), indicating that AMR represents an important national, as well as global, health challenge [2].

In Croatia, as elsewhere, community antibiotic use is particularly relevant to AMR, as the majority of antibacterial consumption occurs outside hospital settings. Across countries with available data, antibacterials dispensed in the community account for approximately 85–95% of total human antibacterial consumption [3,4]. Antibiotic prescribing in primary care contributes to resistance at both individual and population levels. For this reason, surveillance of outpatient antibiotic prescribing is a key component of antimicrobial stewardship [5,6]. The World Health Organization (WHO) Global Action Plan on AMR and the AWaRe framework emphasize the importance of monitoring antibiotic use, promoting the use of Access antibiotics while limiting unnecessary prescribing of broader-spectrum Watch and Reserve agents [7,8]. In Croatia, a national policy framework was established through the National Control Program for Antimicrobial Resistance (2017–2021), covering the early part of the study period [9].

Before the COVID-19 pandemic, many high-income countries reported declining community antibiotic consumption, reflecting stewardship efforts and growing awareness of inappropriate prescribing [10]. The COVID-19 pandemic, declared by WHO on 11 March 2020, substantially disrupted healthcare delivery, healthcare-seeking behavior, and patterns of infectious disease transmission. In many settings, outpatient antibiotic use declined during 2020 and subsequently rebounded in later years [11,12,13]. At the EU/EEA level, ECDC has reported a statistically significant increase in community antibacterial consumption between 2020 and 2024 [4].

Croatia appears to follow a similar pattern. According to the 2024 ECDC country factsheet, total human antibiotic consumption increased from 18.8 defined daily doses (DDD) per 1000 inhabitants per day in 2019 to 21.2 in 2023, while the proportion of Access antibiotics decreased from 62.7% to 60.7%, remaining below the 2030 target of 65% [4].

Detailed long-term analyses of outpatient prescriptions by antibiotic class and in-dividual substance in Croatia are lacking. It is important to describe the volume of overall prescribing but also the potential changes in broader-spectrum vs narrow-spectrum, which is relevant for antimicrobial stewardship and resistance control.

Therefore, we aimed to analyze national outpatient antibiotic prescriptions issued in Croatian primary healthcare between 2015 and 2024 and to identify changes in prescribing trends by antibiotic class and substance.

## 2. Methods

A retrospective analysis of nationwide data on antibiotic prescriptions issued in primary care outpatient settings was conducted covering the period from 1 January 2015, to 31 December 2024. All prescriptions for antibiotics classified under Anatomical Therapeutic Chemical (ATC) code group J01 (antibacterial for systemic use) were included. Antibacterial classes correspond to ATC level 3 (e.g., penicillins = J01C, macrolides = J01F, cephalosporins = J01D, and quinolones = J01M). Individual substances correspond to ATC level 5.

The data were obtained from the Central Health Information System of the Republic of Croatia (CEZIH), a nationwide electronic health information system that captures all prescriptions issued in primary healthcare. The datasets included structured information on e-prescriptions issued in primary care [14]. Primary care in Croatia encompasses family medicine, gynecology and obstetrics, dentistry, and pediatrics. The data from all these sources were included in the analysis.

The primary outcome was the annual number of issued outpatient antibiotic prescriptions, described overall and by antibiotic class and individual substance. The proportions of issued prescriptions for a class or a substance were calculated as percentages of total annual issued prescriptions of antibiotic in outpatient use.

To facilitate the comparison of trends across substances with markedly different prescribing volumes, annual prescription counts for individual substances were additionally expressed as a percentage relative to the 2015 baseline, with 2015 designated as the index year (index = 100%). For presentation, the substances were grouped into three categories based on the direction and magnitude of change relative to 2015 by the end of the study period: substances showing a net decrease below 100% of baseline; substances showing a net increase greater than 30% above the baseline; and substances showing a net change between 0% and 30% above the baseline. The 30% threshold was defined a priori to distinguish substances with a marked increase from those with relatively stable prescribing volume. These groupings are descriptive and do not represent statistical categories.

Antibiotic prescriptions were additionally classified according to the WHO Access, Watch, and Reserve (AWaRe) framework. The annual proportion of Access antibiotic prescriptions was calculated and compared with the WHO target of at least 65% of total antibiotic consumption originating from the Access group [8].

## 3. Results

Between 2015 and 2024, a total of 31,048,414 outpatient antibiotic prescriptions were issued in Croatian primary healthcare.

### 3.1. Distribution of Outpatient Antibiotic Prescriptions by Class and Substance

Table 1 presents the cumulative distribution of outpatient antibiotic prescriptions by antibiotic class and substance in Croatia over the 10-year study period (2015–2024).

Penicillins were the most frequently prescribed class, accounting for 44.29% of all prescriptions (13,752,142 in total). Within this class, prescribing was heavily dominated by co-amoxiclav, which alone accounted for 73.45% of all penicillin prescriptions and 32.53% of all outpatient antibiotic prescriptions issued over the study period. Amoxicillin represented 22.63% of penicillin prescriptions, while phenoxymethylpenicillin (penicillin V) accounted for only 3.81% of penicillin prescriptions. Macrolides were the second most prescribed class, comprising 20.17% of all prescriptions (6,260,985 in total), with azithromycin representing 84.42% of all macrolide prescriptions and 17.02% of all outpatient antibiotic prescriptions. Cephalosporins ranked third at 13.50%, with cefuroxime being the most prescribed substance within this class (43.52%), followed by cephalexin (27.19%), and third-generation cephalosporins (cefixime and cefpodoxime combined; 29.30%). Quinolones accounted for 7.23% of all prescriptions, with ciprofloxacin and norfloxacin together comprising 85.79% of quinolone prescriptions. Among the remaining antibiotic substances, nitrofurantoin was the most frequently prescribed (4.86% of all prescriptions), followed by clindamycin (3.15%), trimethoprim/sulfamethoxazole (2.74%), doxycycline (2.36%), and fosfomycin (1.70%).

It should be noted that co-amoxiclav and azithromycin together accounted for 49.55% of all outpatient antibiotic prescriptions issued in Croatia over the 10-year study period.

### 3.2. Overall Trends in Outpatient Antibiotic Prescribing

**Figure 1:**
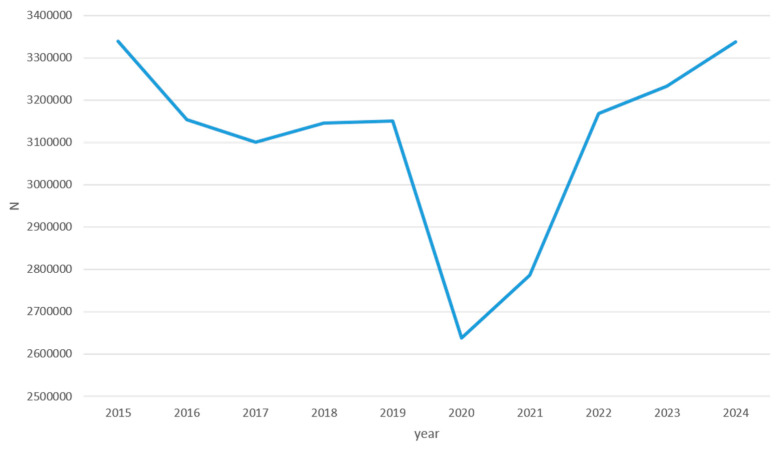
Total number of outpatient antibiotic prescriptions issued in Croatia, 2015–2024. y-axis = number of issued prescriptions, x-axis = years.

Figure 1 illustrates the overall trend in the total number of outpatient antibiotic prescriptions issued in Croatia over the study period (2015–2024).

The total number of outpatient antibiotic prescriptions issued in Croatia showed a slow and gradual decline during the pre-pandemic period, decreasing from 3,339,098 in 2015 to 3,150,919 in 2019—a 5.6% reduction over 4 years. This was followed by a sharp drop in 2020, when prescriptions fell to 2,638,418, representing a decrease of 16.3% in a single year and a 21.0% decline compared with 2015. A progressive recovery began in 2021 (2,786,652 prescriptions) and continued through 2022 (3,168,582) and 2023 (3,233,091), ultimately reaching 3,338,235 prescriptions in 2024—a level virtually identical to that recorded in 2015. By 2024, outpatient antibiotic prescribing had thus fully returned to pre-pandemic levels.

The annual number of issued outpatient antibiotic prescriptions for each substance across the full study period are provided in Supplementary Table S1.

**Figure 2:**
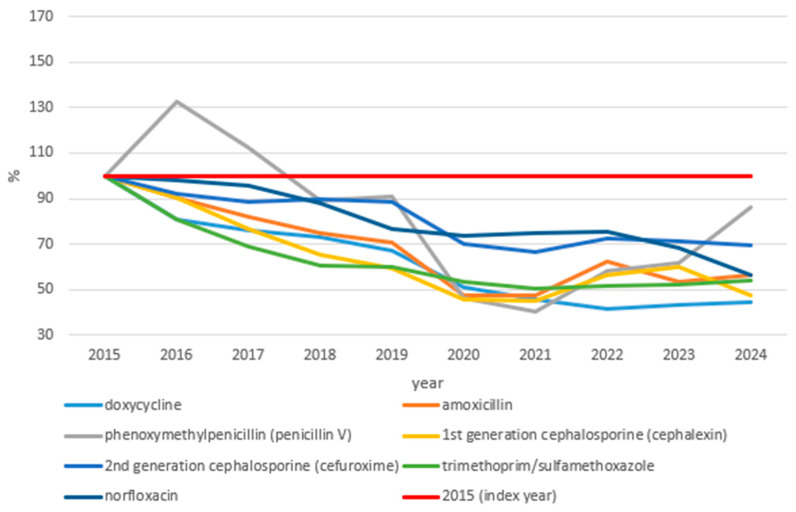
Substances with decreasing prescribing trends (net change below 0% relative to 2015 baseline by 2024). The *y*-axis represents the number of issued prescriptions expressed as a percentage of the 2015 baseline (index year = 100%, shown as the red line).

Figure 2 shows index trends in issued outpatient antibiotic prescriptions for substances with decreasing prescribing trends in Croatia over the study period (2015–2024; 2015 = 100%).

A total of seven substances exhibited declining trends relative to the 2015 levels, all falling below the index value of 100% by 2024. The steepest declines were observed for doxycycline (44.72%), cephalexin (47.34%), and trimethoprim/sulfamethoxazole (53.89%), the latter showing a particularly sharp early decline between 2015 and 2018 before the rate of decrease slowed considerably. Amoxicillin and norfloxacin showed intermediate declines, reaching 56.41% and 56.62% of the 2015 levels by 2024, respectively. Cefuroxime declined more moderately, ending at 69.21% of the baseline. Phenoxymethylpenicillin showed a distinct trajectory, with a notable increase in 2016 briefly exceeding 130% of the baseline, followed by a steady decline through the remainder of the study period, reaching 85.92% of the baseline by 2024, the smallest overall decline among substances in this group. A transient further dip was observed across most substances in 2020, consistent with the overall reduction in antibiotic prescribing during the COVID-19 pandemic, followed by a partial rebound in subsequent years, though none of the substances in this group returned to their 2015 baseline levels.

**Figure 3:**
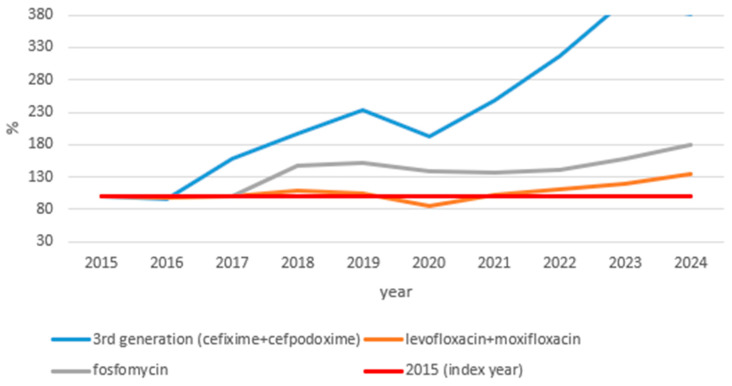
Index graph of issued outpatient antibiotic prescriptions for substances with markedly increasing prescribing trends, Croatia 2015–2024 (2015 = 100%). Substances with a net change >30% relative to the 2015 baseline by 2024 are shown. The *y*-axis represents the number of issued prescriptions expressed as a percentage of the 2015 baseline (index year = 100%, shown as the red line).

Figure 3 shows index trends in issued outpatient antibiotic prescriptions for substances with marked increases over the study period (2015–2024; 2015 = 100%).

A total of three substances exhibited marked increases in prescribing relative to the 2015 levels. The most dramatic rise was observed for third-generation cephalosporins (cefixime and cefpodoxime combined), which increased progressively throughout the study period, briefly declining in 2020, before accelerating sharply, reaching 381.92% of the 2015 levels by 2024—nearly a fourfold increase. Fosfomycin also showed a substantial and largely continuous increase, reaching 179.99% of baseline by 2024, with only a modest attenuation in 2020. Levofloxacin and moxifloxacin combined rose to 135.37% of 2015 levels by 2024, with a notable dip in 2020 followed by a steady post-pandemic recovery.

**Figure 4:**
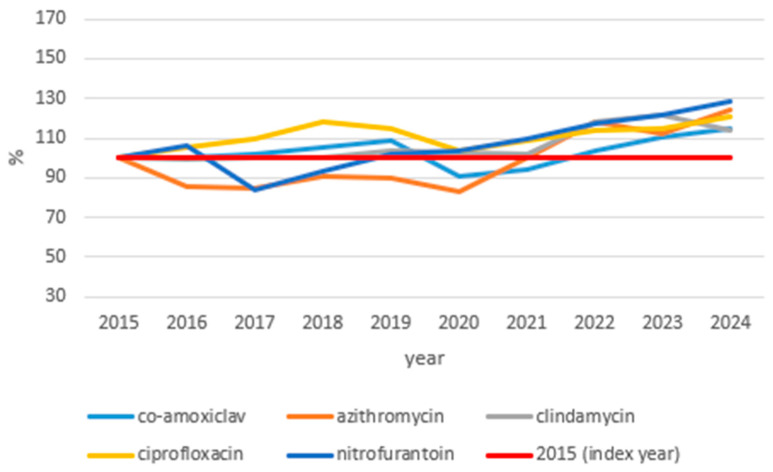
Index graph of issued outpatient antibiotic prescriptions for substances with stable or moderately increasing prescribing trends, Croatia 2015–2024 (2015 = 100%). Substances with a net change between 0% and 30% relative to the 2015 baseline by 2024 are shown. The *y*-axis represents the number of issued prescriptions expressed as a percentage of the 2015 baseline (index year = 100%, shown as the red line).

Figure 4 shows index trends in issued outpatient antibiotic prescriptions for substances with stable or moderately increasing trends over the study period (2015–2024; 2015 = 100%).

Five substances maintained relatively stable or moderately increasing prescribing patterns throughout the study period, ending between 114% and 129% of 2015 levels by 2024. Despite this overall stability, a consistent pattern was observed across all substances: a transient decline in 2020 during the COVID-19 pandemic, followed by recovery and gradual increase in subsequent years. Nitrofurantoin showed the largest increase, reaching 128.30% of the baseline by 2024, with a largely continuous upward trajectory across the study period. Azithromycin and ciprofloxacin reached 123.86% and 120.85% of the 2015 levels, respectively, by 2024, both exhibiting a notable post-pandemic rebound after their respective 2020 dips. Co-amoxiclav and clindamycin showed the most stable overall trajectories, ending at 114.72% and 114.01% of the baseline, respectively, with fluctuations largely limited to the pandemic period.

The calculated index values for all substances are provided in Supplementary Table S2.

### 3.3. AWaRe Classification of Outpatient Antibiotic Prescriptions

**Figure 5:**
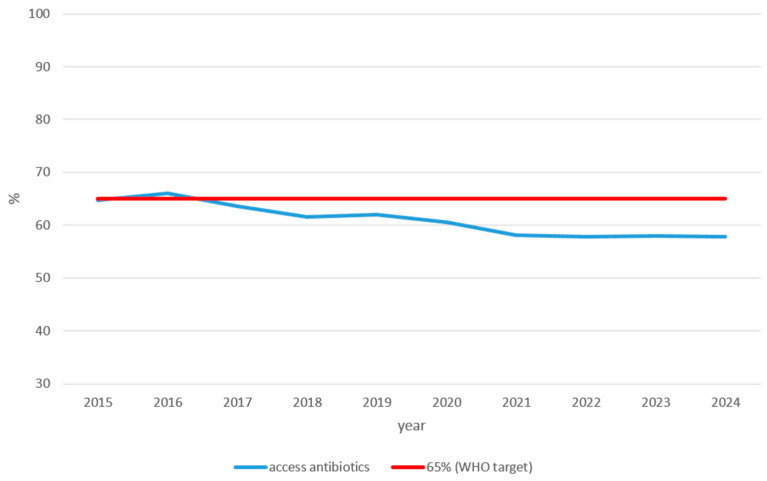
Proportion of outpatient antibiotic issued prescriptions classified as Access antibiotics according to WHO AWaRe classification, Croatia 2015–2024. The *y*-axis represents the percentage of total outpatient antibiotic prescriptions classified as Access antibiotics according to the WHO AWaRe classification. The solid red line indicates the WHO target of at least 65% of total antibiotic consumption being from the Access group.

Figure 5 shows the proportion of issued outpatient antibiotic prescriptions classified as Access antibiotics according to the WHO AWaRe classification in Croatia over the study period (2015–2024).

The proportion of Access antibiotics exhibited an overall declining trend throughout the study period. After briefly reaching 66.09% in 2016—the only year in which Croatia met the WHO target of at least 65% Access antibiotics—the proportion declined consistently, falling from 64.74% in 2015 to 57.88% in 2024. The steepest decline occurred between 2019 and 2021, when the Access proportion fell from 61.94% to 58.12%. From 2021 onwards, the proportion remained relatively stable but consistently below the WHO target, ending at 57.88% in 2024.

## 4. Discussion

### 4.1. Trends in Outpatient Antibiotic Prescriptions Issued in Croatian Primary Healthcare

This study provides a national analysis of trends in outpatient antibiotic prescriptions issued in Croatian primary healthcare over a 10-year study period (2015–2024), spanning the COVID-19 pandemic. Three key findings emerge. First, the overall prescribing declined gradually before the pandemic, dropped sharply in 2020, and fully rebounded by 2024, a pattern consistent with trends observed across the European Union. Second, prescribing throughout the entire period was dominated by broad-spectrum agents, with co-amoxiclav and azithromycin together accounting for approximately half of all prescriptions issued. Third, the composition of prescribing shifted unfavorably over time, with marked increases in Watch-classified antibiotics—particularly third-generation cephalosporins—alongside declines in narrower-spectrum Access agents, resulting in sustained failure to meet the WHO target of 65% Access antibiotic prescribing.

Although total prescribing declined before the COVID-19 pandemic and subsequently returned to pre-pandemic levels by 2024, broad-spectrum antibiotics remained dominant. Co-amoxiclav and azithromycin together accounted for 49.55% of all prescriptions, alongside a more than threefold increase in third-generation cephalosporins. This rebound mirrors European trends, where antibiotic use declined in 2020 and increased again, as healthcare utilization and circulation of respiratory infections returned to pre-pandemic levels [11,15,16,17] but with an unfavorable shift in prescribing composition.

A particular concern is the persistent dominance of co-amoxiclav, which accounted for 73.45% of all penicillin prescriptions and 32.53% of all outpatient antibiotic prescriptions. Although penicillins are largely classified as Access antibiotics, prescribing within this class was heavily skewed toward co-amoxiclav—the broad-spectrum option—with narrower-spectrum alternatives such as amoxicillin and penicillin V underused despite being recommended by guidelines as outpatient first-line agents for the most common infections [18]. Phenoxymethylpenicillin accounted for only 3.81% of penicillin prescriptions, highlighting a persistent preference for broader-spectrum therapy, a recognized marker of suboptimal prescribing quality in European surveillance [19,20].

The increase in third-generation cephalosporin prescribing is particularly concerning. Classified as Watch antibiotics, these agents are generally not recommended as first-line therapy for most common outpatient infections [8,21], yet their prescriptions more than tripled relative to the 2015 levels, reaching 381.92% of the baseline by 2024. Together with the stable high volume of co-amoxiclav and azithromycin, this indicates a prescribing pattern characterized by a persistent preference for broader-spectrum agents.

Macrolide prescribing further supports this pattern. Azithromycin, as a Watch antibiotic, remained the second most prescribed antibiotic throughout the study period, representing 17.02% of all prescriptions and 84.42% of all macrolide use. Its post-pandemic increase may reflect its widespread empirical use in respiratory infections despite limited evidence of benefit for viral etiologies [22,23].

Quinolone trends were mixed. Declines in norfloxacin, alongside increases in nitrofurantoin (128.30% of 2015 levels by 2024) and fosfomycin (179.99%) use, suggest partial improvement in urinary tract infection management, consistent with guideline recommendations favoring these agents over fluoroquinolones as first-line therapy [24]. However, ciprofloxacin remained widely used, and respiratory quinolones increased to 135.37% of baseline, indicating that reduction in fluoroquinolone use remains incomplete. This is particularly relevant given European Medicines Agency recommendations to restrict fluoroquinolone use in mild, uncomplicated infections due to serious adverse effects and their Watch classification reflecting resistance potential [25,26].

These substance-level patterns are directly reflected in the AWaRe trends. Access antibiotics declined from 64.74% in 2015 to 57.88% in 2024, remaining below the WHO target of 65% throughout the study period, with the single exception in 2016. This decline was driven by two concurrent trends: reduced prescribing of several Access agents—such as amoxicillin (56.41% of 2015 levels by 2024), cephalexin (47.34%), and doxycycline (44.72%)—alongside simultaneous increases in Watch antibiotics, particularly third-generation cephalosporins and azithromycin. Notably, some Watch class increases, such as fosfomycin, are aligned with contemporary UTI guidelines [24], but the overall trajectory indicates a sustained move away from Access group prescribing.

Overall, our findings indicate that outpatient antibiotic prescribing in Croatia is characterized by persistent overuse of broad-spectrum antibiotics and insufficient uptake of narrower-spectrum first-line agents. From a stewardship perspective, interventions should focus on optimizing antibiotic selection within therapeutic classes rather than solely reducing overall prescribing volume. In particular, priority targets include reducing co-amoxiclav use in favor of amoxicillin or penicillin V and addressing the rapid growth of third-generation cephalosporin prescribing.

Future research should focus on understanding the drivers of these prescribing patterns, particularly for respiratory infections, which account for a large proportion of outpatient antibiotic use [27]. Linking prescription data with diagnostic information would allow better assessment of prescribing appropriateness and support more targeted interventions.

### 4.2. Limitations

This study has several limitations. The data reflect prescriptions issued rather than antibiotics actually dispensed or consumed by patients, describing prescribing behavior rather than true antibiotic exposure. The use of aggregated data without indication information prevents the assessment of prescribing appropriateness. Additionally, the dataset includes only primary care prescriptions, while hospital and specialist outpatient prescribing are not captured.

## 5. Conclusions

Throughout the study period, outpatient antibiotic prescribing in Croatian primary healthcare was dominated by broad-spectrum agents, and the proportion of Access antibiotics consistently remained below the WHO target of 65%. These findings indicate that the main challenge for antimicrobial stewardship in Croatia lies not only in overall prescribing volume but in prescribing composition. Targeted interventions to improve antibiotic selection within therapeutic classes—particularly reducing co-amoxiclav use and addressing the rapid growth of third-generation cephalosporins—are needed to align Croatian prescribing patterns with stewardship recommendations.

## Figures and Tables

**Table 1:** Number of outpatient antibiotic prescriptions in Croatia, cumulative data, 2015–2024.

Drugs and Drug Groups	Sum of Prescriptions 2015–2024	% of Prescriptions Within Each Antibiotic Class	% of all Issued Prescriptions of Antibiotics in Outpatient Care
**Penicillins**	13,752,142	n/a	44.29
Co-amoxiclav	10,101,321	73.45	32.53
Amoxicillin	3,112,227	22.63	10.02
Phenoxymethylpenicillin (penicillin V)	524,620	3.81	1.69
Flucloxacillin	13,974	0.10	0.05
**Macrolides**	6,260,985	n/a	20.17
Azithromycin	5,285,728	84.42	17.02
Clarithromycin	942,071	15.05	3.03
Erythromycin	33,186	0.53	0.11
**Cephalosporines**	4,191,915	n/a	13.50
Cefuroxime	1,824,382	43.52	5.88
Cephalexin	1,139,650	27.19	3.67
Cefixime	855,403	20.41	2.76
Cefpodoxime	372,480	8.89	1.20
**Quinolones**	2,245,468	n/a	7.23
Ciprofloxacin	1,035,418	46.11	3.33
Norfloxacin	890,934	39.68	2.87
Levofloxacin	232,656	10.36	0.75
Moxifloxacin	86,460	3.85	0.28
Remaining Antibiotic Substances *			
Nitrofurantoin	1,508,435	n/a	4.86
Clindamycin	978,147	n/a	3.15
Trimethoprim/sulfamethoxazole	849,233	n/a	2.74
Doxycycline	733,085	n/a	2.36
Fosfomycin	529,004	n/a	1.70

n/a = not applicable. * These substances are the sole representatives of their respective ATC antibiotic class in the Croatian outpatient setting and are therefore presented without within-class proportions. Total number of outpatient antibiotic prescriptions issued during the study period: 31,048,414.

## Data Availability

We are not able to make our underlying data set publicly available since the data we used had been collected for the primary purpose of healthcare provision. Our institution’s Ethical Committee approved secondary use of data for research purposes and publishing in form of scientific paper. The raw data can be shared to third parties by submitting the request to our institution, the contact is Department of Biostatistics of Croatian Institute of Public Health (biostatistika@hzjz.hr).

## Supplementary Materials

The following supporting information can be downloaded at: https://www.mdpi.com/article/10.3390/idr18020036/s1, Table S1: Number of outpatient antibiotics prescriptions from 2015 to 2024; Table S2: Index values of outpatient antibiotic prescriptions by class and substance, Croatia 2015–2024 (2015 = 100%).
