# Supplementary material for: Trends in Outpatient Antibiotic Prescriptions Issued in Croatian Primary Healthcare, 2015–2024
Source: Infect Dis Rep. 2026 Apr 14;18(2):36. doi: 10.3390/idr18020036 (PMC13116669; doi:10.3390/idr18020036)
Supplement: Supplementary file 1 [file idr-18-00036-s001.zip › idr-4083123-supplementary.pdf]

Table S1. Number of outpatient antibiotics prescriptions from 2015 to 2024.

	2015	2016	2017	2018	2019	2020	2021	2022	2023	2024
**Penicillins**	**1500172**	**1478830**	**1442459**	**1430557**	**1448153**	**1142111**	**1169794**	**1339146**	**1364913**	**1436772**
amoxicillin	454209	410592	371141	340114	322206	216660	215120	283242	242718	256225
phenoxymethylpenicillin (penicillin V)	64126	85128	72149	57127	58383	29792	26034	37316	39465	55100
flucloxacillin	58	448	778	1154	1572	1959	2479	2625	2901	0
co-amoxiclav	981016	982662	998391	1032162	1065992	893700	926161	1015961	1079829	1125447
**Cephalosporins**	**458974**	**420208**	**417857**	**421970**	**427161**	**340821**	**361842**	**429458**	**478789**	**440738**
1st generation (cephalexin)	176509	159717	135367	115135	104293	80279	80011	99287	105497	83555
2nd generation (cefuroxime)	225397	207877	199426	202731	199788	158525	150548	163431	160654	156005
3rd generation*	52676	51103	83064	104104	123080	102017	131283	166740	212638	201178
cefixime	43798	42080	65104	78124	87719	73701	90459	115598	136845	121975
cefpodoxime	8878	9023	17960	25980	35361	28316	40824	51142	75793	79203
**Macrolides**	**752684**	**660382**	**656073**	**687354**	**680774**	**610553**	**700958**	**828939**	**805010**	**856405**
erythromycin	4954	4221	3712	3782	3228	2719	3438	3460	3672	0
clarithromycin	120560	106574	107491	103505	102896	69511	68185	85823	88869	88657
azithromycin	535024	457843	452751	487813	478939	443669	535791	630599	600610	662689
**Quinolones**	**233697**	**236451**	**237973**	**239623**	**222628**	**202844**	**215430**	**222650**	**218555**	**215781**
ciprofloxacin	93296	98678	101973	109906	107128	96460	101649	106309	107273	112746
norfloxacin	110410	108529	105837	96966	84464	81076	82815	83028	75293	62516
respiratory quinolones**	29933	29207	30140	32738	31020	25297	30963	33310	35989	40519
levofloxacin	18769	19434	19400	21514	21456	18872	24122	26949	29223	32917
moxifloxacin	11164	9773	10740	11224	9564	6425	6841	6361	6766	7602
**Remaining antibiotic substances*** **	**141565**	**154241**	**164613**	**198494**	**213235**	**209946**	**217094**	**230072**	**244661**	**263524**
nitrofurantoin	141565	150158	119108	131651	144454	146776	154653	166125	172324	181621
fosfomycin	0	4083	45504	66843	68781	63170	62438	63946	72336	81903
clindamycin	92146	91744	92119	92254	95711	94654	93544	109057	111859	105059
doxycycline	117736	95166	89215	85795	78803	60339	53492	49100	50785	52654
trimethoprim/sulfamethoxazole	134270	108748	92827	81459	80165	71804	68042	69217	70378	72361
Total	3339098	3154026	3101017	3145252	3150919	2638418	2786652	3168582	3233091	3338235

* cefixime+cefpodoxime

**levofloxacin+moxifloxacin

*** These substances are the sole representative of their respective ATC antibiotic class in the Croatian outpatient setting and are therefore presented without within-class proportions.

Table S2. Index values of outpatient antibiotic prescriptions by class and substance, Croatia 2015–2024 (2015 = 100%)

	2015	2016/2015	2017/2015	2018/2015	2019/2015	2020/2015	2021/2015	2022/2015	2023/2015	2024/2015
Tetracyclines (doxycycline)	100,00	80,82999	75,77546	72,87066	66,93195	51,24941	45,43385	41,70347	43,13464	44,72209
Penicillins	100,00	98,57736	96,15291	95,35953	96,53246	76,132	77,97733	89,26616	90,98377	95,77382
amoxicillin	100,00	90,39715	81,7115	74,88051	70,93783	47,70051	47,36146	62,3594	53,43751	56,41126
phenoxymethylpenicillin (penicillin V)	100,00	132,7511	112,5113	89,08555	91,04419	46,45853	40,5982	58,19169	61,5429	85,92459
flucloxacillin	100,00	772,4138	1341,379	1989,655	2710,345	3377,586	4274,138	4525,862	5001,724	0
co-amoxiclav	100,00	100,1678	101,7711	105,2136	108,662	91,09943	94,40835	103,5621	110,0725	114,7226
Cephalosporins	100,00	91,55377	91,04154	91,93767	93,06867	74,25715	78,83715	93,56913	104,3172	96,02679
1st generation (cephalexin)	100,00	90,4866	76,69127	65,22897	59,08651	45,48153	45,3297	56,25039	59,76862	47,33753
2nd generation (cefuroxime)	100,00	92,22705	88,47766	89,94397	88,63827	70,33146	66,79237	72,50806	71,27602	69,21343
3rd generation*	100,00	97,01382	157,6885	197,6308	233,6548	193,6688	249,2274	316,5388	403,6715	381,9159
Cefixime	100,00	96,07745	148,6461	178,3734	200,2808	168,2748	206,5368	263,9344	312,4458	278,4945
cefpodoxime	100,00	101,6333	202,2978	292,6335	398,2992	318,9457	459,8333	576,0532	853,7171	892,1266
Sulfonamides and trimethoprim (trimethoprim/sulfamethoxazole)	100,00	80,99203	69,13458	60,66806	59,70433	53,47732	50,6755	51,55061	52,41528	53,89216
Macrolides	100,00	87,73695	87,16447	91,32039	90,44619	81,11678	93,12779	110,1311	106,9519	113,7802
erythromycin	100,00	85,20388	74,92935	76,34235	65,15947	54,88494	69,39847	69,84255	74,12192	0
clarithromycin	100,00	88,39914	89,15975	85,85352	85,34837	57,65677	56,5569	71,18696	73,7135	73,53766
azithromycin	100,00	85,57429	84,62256	91,17591	89,51729	82,92507	100,1434	117,8637	112,2585	123,8615
clindamycin	100,00	99,56374	99,9707	100,1172	103,8689	102,7218	101,5172	118,3524	121,3932	114,0136
Quinolones	100,00	101,1784	101,8297	102,5358	95,26352	86,79786	92,18347	95,27294	93,52067	92,33366
ciprofloxacin	100,00	105,7687	109,3005	117,8035	114,8259	103,3914	108,9532	113,9481	114,9813	120,8476
norfloxacin	100,00	98,29635	95,85817	87,82357	76,50032	73,43175	75,00679	75,19971	68,194	56,62168
Respiratory quinolones**	100,00	97,57458	100,6915	109,3709	103,6314	84,51208	103,441	111,2819	120,2319	135,3656
Levofloxacin	100,00	103,5431	103,3619	114,6252	114,3162	100,5488	128,5204	143,5825	155,6982	175,3796
Moxifloxacin	100,00	87,54031	96,20208	100,5374	85,66822	57,55106	61,27732	56,97779	60,60552	68,09387
Other antibacterials	100,00	108,9542	116,2809	140,214	150,6269	148,3036	153,3529	162,5204	172,8259	186,1505

Nitrofurantoin	100,00	106,07	84,13662	92,99686	102,0408	103,681	109,2452	117,3489	121,7278	128,2951
fosfomycin	0	0	100,00	146,8948	151,1537	138,823	137,2143	140,5283	158,9662	179,9908
Total	100	94,45743	92,8699	94,19466	94,36438	79,01589	83,45523	94,89335	96,82528	99,97415

Each value represents the number of issued outpatient antibiotic prescriptions in a given year expressed as a percentage of the 2015 baseline (index year = 100%). Values above 100% indicate an increase relative to 2015; values below 100% indicate a decrease. Fosfomycin was introduced into the Croatian outpatient formulary during the study period and 2017 was therefore used as its index year. Flucloxacillin data for 2024 are not available. Abbreviations: * cefixime + cefpodoxime combined; ** levofloxacin + moxifloxacin combined.
